# The Defense Response Involved in Sweetpotato Resistance to Root-Knot Nematode *Meloidogyne incognita*: Comparison of Root Transcriptomes of Resistant and Susceptible Sweetpotato Cultivars With Respect to Induced and Constitutive Defense Responses

**DOI:** 10.3389/fpls.2021.671677

**Published:** 2021-05-05

**Authors:** Il-Hwan Lee, Ho Soo Kim, Ki Jung Nam, Kang-Lok Lee, Jung-Wook Yang, Sang-Soo Kwak, Jeung Joo Lee, Donghwan Shim, Yun-Hee Kim

**Affiliations:** ^1^Department of Forest Bio-Resources, National Institute of Forest Science, Suwon, South Korea; ^2^Plant Systems Engineering Research Center, Korea Research Institute of Bioscience and Biotechnology (KRIBB), Daejeon, South Korea; ^3^Department of Biology Education, IALS, Gyeongsang National University, Jinju, South Korea; ^4^Department of Crop Cultivation & Environment, Research National Institute of Crop Science, Rural Development Administration, Suwon, South Korea; ^5^Department of Plant Medicine, IALS, Gyeongsang National University, Jinju, South Korea; ^6^Department of Biological Sciences, Chungnam National University, Daejeon, South Korea

**Keywords:** constitutive defense, induced defense response, resistant cultivars, root-knot nematodes, susceptible cultivar, sweetpotato, transcriptome

## Abstract

Sweetpotato (*Ipomoea batatas* [L.] Lam) is an economically important, nutrient- and pigment-rich root vegetable used as both food and feed. Root-knot nematode (RKN), *Meloidogyne incognita*, causes major yield losses in sweetpotato and other crops worldwide. The identification of genes and mechanisms responsible for resistance to RKN will facilitate the development of RKN resistant cultivars not only in sweetpotato but also in other crops. In this study, we performed RNA-seq analysis of RKN resistant cultivars (RCs; Danjami, Pungwonmi and Juhwangmi) and susceptible cultivars (SCs; Dahomi, Shinhwangmi and Yulmi) of sweetpotato infected with *M. incognita* to examine the induced and constitutive defense response-related transcriptional changes. During induced defense, genes related to defense and secondary metabolites were induced in SCs, whereas those related to receptor protein kinase signaling and protein phosphorylation were induced in RCs. In the uninfected control, genes involved in proteolysis and biotic stimuli showed differential expression levels between RCs and SCs during constitutive defense. Additionally, genes related to redox regulation, lipid and cell wall metabolism, protease inhibitor and proteases were putatively identified as RKN defense-related genes. The root transcriptome of SCs was also analyzed under uninfected conditions, and several potential candidate genes were identified. Overall, our data provide key insights into the transcriptional changes in sweetpotato genes that occur during induced and constitutive defense responses against RKN infection.

## Introduction

Sweetpotato (*Ipomoea batatas* [L.] Lam) is the fifth most important food crop in the world and a representative root vegetable, with a total production of 91.8 million tons worldwide and an annual harvest area of 7.7 million ha. Sweetpotato is cultivated primarily in Asia and Africa (Afuape et al., 2014; Heider et al., 2021), where it plays an important role in sustainable agriculture, as it serves as a valuable source of nutrients, including minerals, vitamins and pigments, as well as processed foods, animal feeds and alcohol (Diaz et al., 2014; Grace et al., 2014). However, given the narrow genetic base of cultivated sweetpotato, together with its complex hexaploid genome, the development of sweetpotato cultivars with pathogen resistance, high yield, high quality and other desirable traits remains challenging.

Sweetpotato production around the world is affected by various pathogens, including viruses, fungi and parasitic nematodes (Clark and Moyer, 1998; Kreuze, 2002; Palomares-Rius and Kikuchi, 2013). Among the plant parasitic nematodes, root-knot nematodes (RKNs), members of the genus *Meloidogyne*, represent a major threat to many agricultural crops including sweetpotato (Castagnone-Sereno et al., 2013; Kim and Yang, 2019). *Meloidogyne incognita* is a destructive RKN and the most common nematode species found in agricultural regions worldwide. Sweetpotato is highly susceptible to RKN, especially *M. incognita*, which occurs in tropical regions throughout the world and causes severe damage to plant roots (Bridge and Starr, 2010; Kim and Yang, 2019). However, studies on the mechanism of resistance to RKN in sweetpotato are still lacking.

To respond to pathogen infection, including plant parasitic nematode infestation, host plants employ both constitutive and induced defense mechanisms (Boots and Best, 2018). Constitutive defense prevents infection in the first place, while induced defense typically shortens the infectious period. Therefore, these two defense routes have very different implications, not only in individuals but also in terms of the epidemiology of the disease. Moreover, the cost of constitutive defense mechanisms is likely to be paid even in the absence of the pathogen, while induced defense is likely to incur the most substantial cost when used in response to pathogen infection (Lam et al., 2001; Wittstock and Gershenzon, 2002). However, there has been no research on the inducible and constitutive defense mechanisms of host plants under nematode attack.

Sweetpotato cultivars show differences in their susceptibility and resistance to RKNs, which can be exploited to reveal the genes and mechanisms responsible for resistance to RKN. We previously conducted proteome and transcriptome profiling of two sweetpotato cultivars, Yulmi and Juhwangmi, with contrasting responses to infection with *M. incognita* (Ha et al., 2017; Lee et al., 2019). Recently, we identified and studied the responses of candidate genes to RKN infection in these two cultivars (Sung et al., 2019; Lee et al., 2020). However, these studies were limited in their ability to elucidate the mechanism of RKN resistance in sweetpotato because the analysis was limited to only two cultivars, and the late response to RKN (generation of number of galls) was confirmed after 2 months in the growth chamber and 3 months in the greenhouse. In the experimental conditions of this study, transcriptome analysis was performed at 1 week after RKN egg inoculation to confirm the early response, and the numbers of galls produced by RKN were counted at 4 weeks from the eggs of nematodes. Therefore, in the present study, considering that RKN infection is a major limiting factor affecting sweetpotato production, we aimed to decipher the RKN resistance mechanism of sweetpotato by performing RNA-seq analysis of three susceptible cultivars (SCs; Dahomi, Shinhwangmi and Yulmi) and three resistant cultivars (RCs; Danjami, Pungwonmi and Juhwangmi) of sweetpotato infected with *M. incognita*, and examined the induced and constitutive defense response-related transcriptional changes in these cultivars. The results revealed transcriptional changes in genes involved in defense response, secondary metabolites, cellular response and macromolecule metabolism during induced defense response. During constitutive defense, genes related to redox regulation, lipid and cell wall metabolism, protease inhibitor and proteases were putatively identified as RKN resistance genes in RCs. Several downregulated potential candidate genes involved in regulations of metabolism, signal and cell wall were also identified in SCs, based on their transcriptome analysis under uninfected root conditions.

## Materials and Methods

### Plant Materials and *M. incognita* Treatment

Six sweetpotato (*Ipomoea batatas* [L.] Lam) cultivars obtained from the Bioenergy Crop Research Center, National Crop Research Institute (RDA, Muan, Jeonnam, Korea) were used in this study; these included RKN sensitive cultivars (SCs), namely Dahomi (DHM), Shinhwangmi (SHM) and Yulmi (YM), and RKN resistant cultivars (RCs), including Danjami (DJM), Pungwonmi (PWM), and Juhwangmi (JHM). The six cultivars used in this study had different origins. Among RCs, DJM was the result of a cross between Yeonjami and Yeonhwangmi. PWM was the result of a cross between benisazma and Luby3074, and JHM was the result of a cross between SQ27 and BB95024-2. Among SCs, DHM was the result of a cross between Muan-4 and Jinhongmi. SHM was the result of a cross between MI874-1 and Ddosabeni, and YM was the result of a cross between Jinmi and MI78001-15. Fifteen plants per sweetpotato variety were planted in sterilized sand: soil mixture (50:50) in perforated 500-cm^3^ clay pots arranged in a completely randomized design. The pots were placed in a greenhouse maintained at 25 ± 3°C, and plants were watered as required. Two weeks after planting, approximately 3,000 *M. incognita* eggs were applied to the soil in each pot and covered with a moist layer of sand. Inoculated and uninoculated plants were harvested 1 week, and the number of galls was visually rated by staining with 0.015% Phloxin B solution for 15 min in roots harvested at 4 weeks after inoculation, as described previously (Fassuliotis, 1985). For each cultivar, seven root samples were ground to a fine powder in liquid nitrogen using a pestle and mortar, and stored at –70°C until needed for further analysis.

### RNA Extraction, cDNA Library Construction, and Sequencing

Total RNA was isolated from fibrous sweetpotato roots using TRIzol RNA Isolation Kit (Invitrogen, United States). Samples with an RNA integrity number (RIN) > 8 were used for library construction. Each paired-end cDNA library was prepared according to the TruSeq RNA Sample Preparation Guide (Illumina, San Diego, CA, United States) and then sequenced on the HiSeq 2500 platform. Three independent replications were performed for each sample.

### RNA-Seq Data Analysis

Paired-end reads were cleaned using prinseq-lite version 0.20.4, with the following parameters: min_len 50; min_qual_score 5; min_qual_mean 20; derep 14; trim_qual_left 20; trim_qual_right 20. Clean paired-end reads of each sample were aligned to the sweetpotato reference genome sequence^1^ using Bowtie2. The RSEM 1.3.0 software was used to obtain read counts and TMM-normalized TPM (trimmed mean of M value-normalized transcripts per million) values for each transcript. EdgeR version 3.16.5 was used to calculate the negative binomial dispersion across conditions for differential gene expression analysis. Genes were determined to be significantly differentially expressed if they showed >4-fold change in expression, with a false discovery rate (FDR)-adjusted *P* < 0.001. Principal component analysis (PCA) plot and heatmap analysis was utilized to visualize and assess the clustering of the data using programs of Mev and PtR of Trinity package (Howe et al., 2011; Haas et al., 2013; Braich et al., 2019).

### Functional Annotation

Functional annotation of differentially expressed genes (DEGs) was performed via sequence similarity searches using the BLAST program against the *Arabidopsis thaliana* protein database, with an *e*-value threshold of 1E-5. Gene Ontology (GO) term and Kyoto Encyclopedia of Genes and Genomes (KEGG) pathway enrichment analyses were performed using DAVID^2^. To conduct MapMan analysis, *Arabidopsis* homolog gene IDs and fold changes of DEGs in the six sweetpotato cultivars were mapped to biotic stress pathways. Pictorial representations of the biotic stress pathways were uploaded from the MapMan website^3^.

### Quantitative Real-Time PCR (qRT-PCR)

Gene expression was verified by qRT-PCR analysis in a fluorometric thermal cycler (DNA Engine Opticon 2; MJ Research, Waltham, MA, United States) using gene-specific primers (Supplementary Table 1) and EvaGreen fluorescent dye, according to the manufacturer’s instructions. Data were normalized relative to the mean CT value of the stable reference gene, *ADP-ribosylation factor* (*ARF*) (Livak and Schmittgen, 2001; Park et al., 2012).

### Statistical Analyses

Data were analyzed by one-way analysis of variance (ANOVA). Statistical significance levels were set at *P* < 0.05. The subsequent multiple comparisons were examined based on Dunnett’s multiple range test. All statistical analyses were performed using Statistical Package for the Social Sciences software (SPSS 12).

## Results

### RKN Resistance Differs Between Sweetpotato Cultivars

**FIGURE 1:**
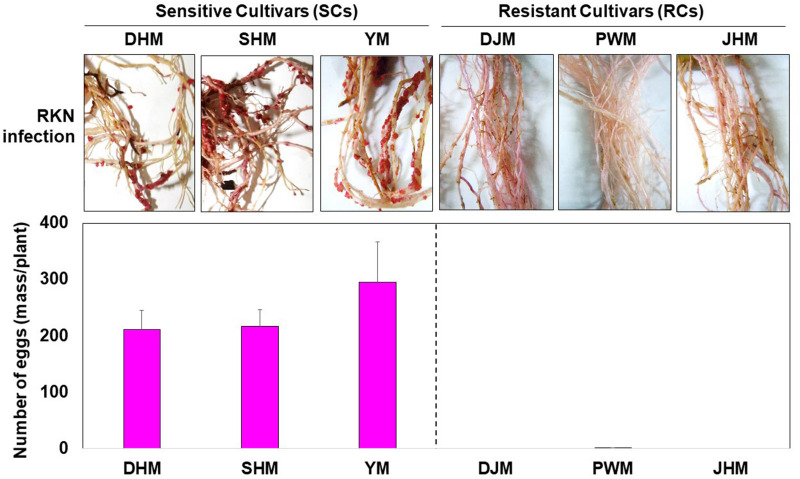
Effect of the root-knot nematode (RKN) *Meloidogyne incognita* on susceptible cultivars (SCs; DHM, SHM, and YM) and resistant cultivars (RCs; DJM, PWM, and JHM) of sweetpotato. Plants were grown under greenhouse conditions for 1 month. Egg masses were formed in non-sterilized sand containing *M. incognita* but not in sweetpotato plants cultured in sterilized soil. Data represent mean ± standard deviation (SD) of 10 replicates. DHM, Dahomi; SHM, Shinhwangmi; YM, Yulmi; DJM, Danjami; PWM, Pungwonmi; JHM, Juhwangmi.

According to the Bioenergy Crop Research Center and our previous study on Korean sweetpotato accessions maintained in South Korea (Choi et al., 2006; Lee et al., 2012; Ha et al., 2017), cultivars DHM, SHM and YM are highly sensitive to RKNs, specifically *M. incognita*, whereas DJM, PWM and JHM are highly resistant. Therefore, we compared the resistance of SCs and RCs to *M. incognita* by measuring nematode egg mass formation after infestation under greenhouse conditions. The experiment was conducted by checking the roots at 1 week (7 days) and 4 weeks (28 days) after infection with RKN eggs. At 1 week after infection, the state of the roots was not significantly changed by RKN infection irrespective of the cultivar. Interestingly, at 4 weeks after infection, galls were found only in susceptible cultivars. *M. incognita* formed 210, 216, and 295 egg masses in DHM, SHM and YM plants, respectively, but only 0–0.6 egg masses in RCs, such as DJM, PWM, and JHM (Figure 1). Thus, these data confirm the differences in *M. incognita* resistance levels between SCs and RCs.

### Transcriptome Sequencing of Sweetpotato Fibrous Roots in Response to RKN

In order to confirm the mechanism of resistance during the early response to RKN infection, transcriptome analysis was performed on roots at 7 days after infection. To analyze the effect of *M. incognita* infection on the transcriptome of fibrous roots of SCs and RCs, we performed RNA-seq analysis of *M. incognita*-infected and uninfected (control) plants using the Illumina HiSeq2500 platform. A total of 693,206,233 raw paired-end reads (140,027,659,066 bp) were generated from the six cultivars. After filtering out low-quality and unpaired reads using the prinseq-lite software, we obtained 612,064,327 high-quality paired-end reads (Supplementary Table 2). All raw read data were deposited at the National Center for Biotechnology Information (NCBI) Sequence Read Archive (SRA) database under the accession number SRP128609 (PRJNA429283).

### Global Statistical Evaluation of Samples Used for Comparative Transcriptomics

**FIGURE 2:**
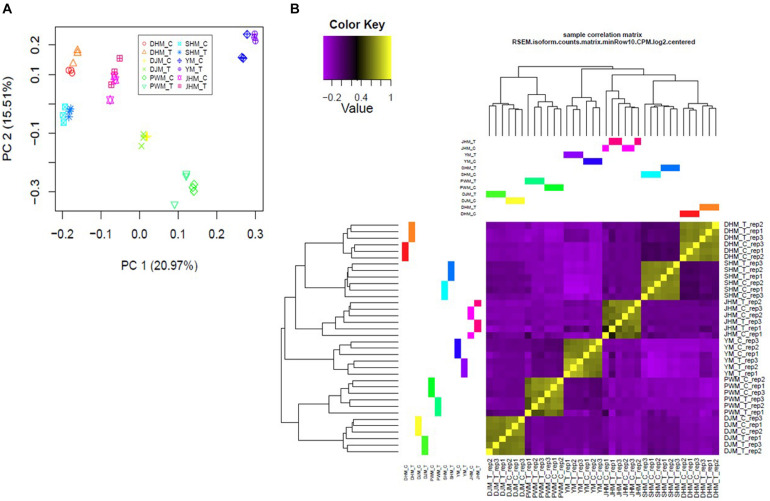
Statistical analysis of differentially expressed genes (DEGs) across samples. **(A)** Principal component analysis (PCA) of all samples by treatment shows general clustering of samples with a few outliers. Colors denote different cultivars and treatment conditions. **(B)** Clustered heatmap showing the Pearson correlation matrix of pairwise sample comparisons. The color key was adjusted based on the log2-centered values for optimal visual detection of differences, and the dendrogram illustrates the relationship among samples. DHM, Dahomi; DJM, Danjami; PWM, Pungwonmi; SHM, Shinhwangmi; JHM, Juhwangmi; YM, Yulmi; RCs, resistant cultivars; SCs, sensitive cultivars; C, control; T, treatment.

To perform a statistical evaluation of samples used for comparative transcriptome analyses, we mapped high-quality reads to the reported transcriptome^4^, and calculated transcript abundance. Next, we performed principal component analysis (PCA) to evaluate the transcriptomic differences among the six sweetpotato cultivars under *M. incognita* treated (T) and control conditions (C) (Figure 2A). In pairwise comparisons, independent biological replicates were more highly correlated within samples than between samples, and biological replicates of a given cultivar clustered with each other than with other cultivars, regardless of the treatment (*M. incognita*-infected or control) (Figure 2B). We also identified DEGs based on pairwise sample comparisons. Each SC and RC showed different patterns of DEG distribution between the control and *M. incognita* infection treatments, as shown by the volcano plots (Supplementary Figure 1). Thus, each of RCs and SCs showed different patterns between untreated and RKN-treated conditions.

### Identification and Characterization of DEGs of the Induced Defense Response

**FIGURE 3:**
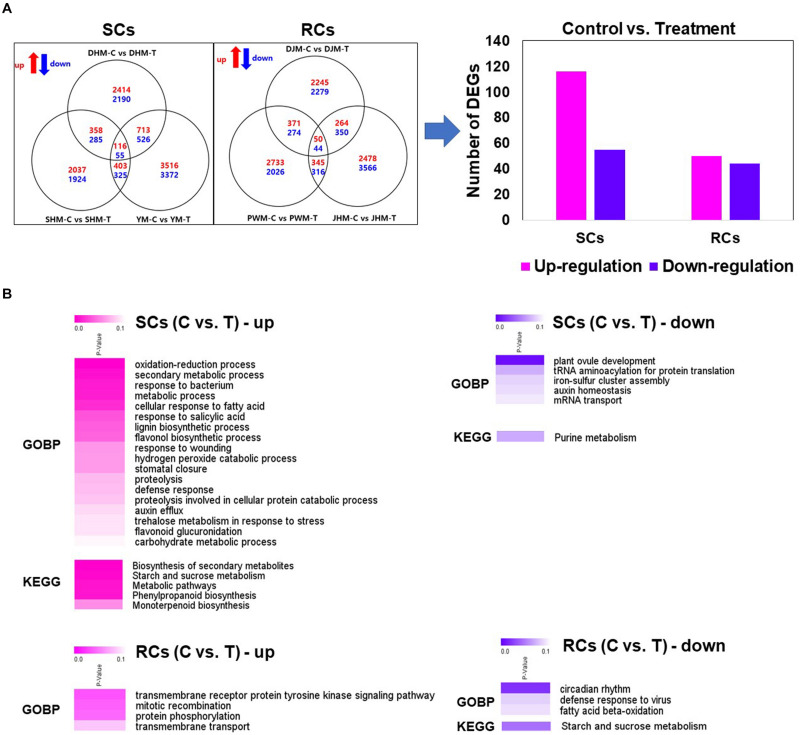
Identification of DEGs in pairwise comparisons across samples, and heatmap of Gene Ontology (GO) biological process category and Kyoto Encyclopedia of Genes and Genomes (KEGG) pathways enriched in the induced defense response. **(A)** Venn diagrams showing the number of DEGs with up- or downregulated expression in the pairwise sample comparisons. **(B)** Heatmap of GO biological process category and KEGG pathway of DEGs in pairwise sample comparisons between control (C) and treatment (T). The heatmap shows Benjamini–Hochberg-adjusted (*P* < 0.05) for DEGs enriched in specific GO terms in the biological process category. DHM, Dahomi; DJM, Danjami; PWM, Pungwonmi; SHM, Shinhwangmi; JHM, Juhwangmi; YM, Yulmi; RCs, resistant cultivars; SCs, sensitive cultivars; C, control; T, treatment.

To analyze the induced defense response of sweetpotato during *M. incognita* infection via RNA-seq, we identified putative unique transcripts as reliable DEGs (fold change > 2; Kal’s *z*-test FDR *P* < 0.005) in pairwise sample comparisons (Figure 3A). First, we examined the transcriptional responses of both SC and RC groups against *M. incognita*. Among the identified DEGs, 116 and 55 were significantly up- and downregulated in SCs, respectively, after *M. incognita* infection compared with the control, consistent with the three cultivars DHM, SHM and YM (Figure 3A and Supplementary Table 3). Fifty upregulated and 44 downregulated DEGs were identified in RCs infected with *M. incognita* compared with the control (Figure 3A and Supplementary Table 4). To functionally characterize the DEGs, we identified their encoded products via comparisons with *A. thaliana* protein database, and then performed GO and KEGG pathway enrichment analyses of the annotated genes (with Benjamini–Hochberg-adjusted *P* < 0.05; Figure 3B). In SCs, several genes differentially expressed between *M. incognita*-infected and control treatments were enriched in the biological process category under various GO terms, including process of oxidation-reduction, secondary metabolic process, response to bacterium, cellular response to fatty acid and response to salicylic acid, and in KEGG pathways including biosynthetic secondary metabolites, starch and sucrose metabolism, phenylpropanoid biosynthesis and monoterpenoid biosynthesis. In RCs, GO terms enriched in the biological process category included transmembrane receptor protein tyrosine kinase signaling pathway, mitotic recombination, and protein phosphorylation with untreated control and RKN infection.

### Differential Regulation of RKN Resistance-Related Candidate Genes Involved in Induced Defense Response

**FIGURE 4:**
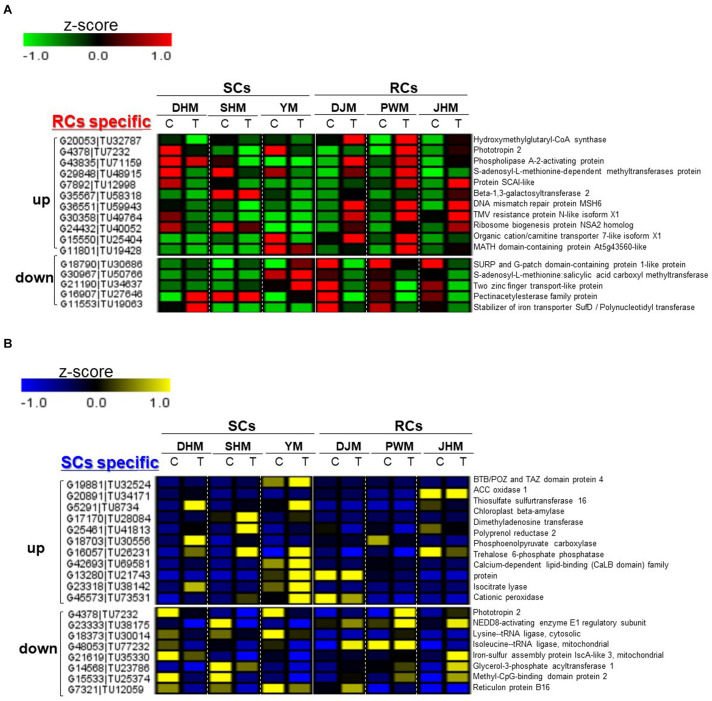
Comparison of the expression levels of induced defense response-related specific candidate genes in six sweetpotato cultivars under RKN-infected and control conditions. DEGs include **(A)** RC-specificand **(B)** SC-specific up- and downregulated genes. DHM, Dahomi; DJM, Danjami; PWM, Pungwonmi; SHM, Shinhwangmi; JHM, Juhwangmi; YM, Yulmi; RCs, resistant cultivars; SCs, sensitive cultivars; C, control; T, treatment. The heatmap was constructed using Multi Experiment Viewer (MeV).

We identified RC- and SC-specific DEGs involved in induced defense response (Figure 4). Among the RC-specific induced defense response-related DEGs (Figure 4A), genes encoding hydroxymethylglutaryl-CoA synthase (G20053| TU32787), phototropin 2 (G4378| TU7232), phospholipase A-2-activating protein (G43835| TU71159) and beta-1,3-galactosyltransferase 2 (G35567| TU58318) were upregulated, whereas those encoding a S-adenosyl-L-methionine: salicylic acid carboxyl methyltransferase (G30967| TU50766), two zinc finger transport-like proteins (G21190| TU34637) and a pectinacetylesterase family protein (G16907| TU27646) were downregulated RKN infection. Among the SC-specific DEGs (Figure 4B), genes encoding ACC oxidase 1 (G20891| TU34171), thiosulfate sulfurtransferase 16 (G5291| TU8734), chloroplast beta-amylase (G17170| TU28084), polyprenol reductase 2 (G18703| TU30556), phosphoenolpyruvate carboxylase (G16057| TU26231), trehalose 6-phosphate phosphatase (G42693| TU69581) and cationic peroxidase (G45573| TU73531) were upregulated, whereas those encoding phototropin 2 (G4378| TU7232), isoleucine–tRNA ligase (G48053| TU77232) and glycerol-3-phosphate acyltransferase 1 (G14568| TU23786) were downregulated.

### Expression Profiling of RKN-Responsive Candidate Genes by qRT-PCR

**FIGURE 5:**
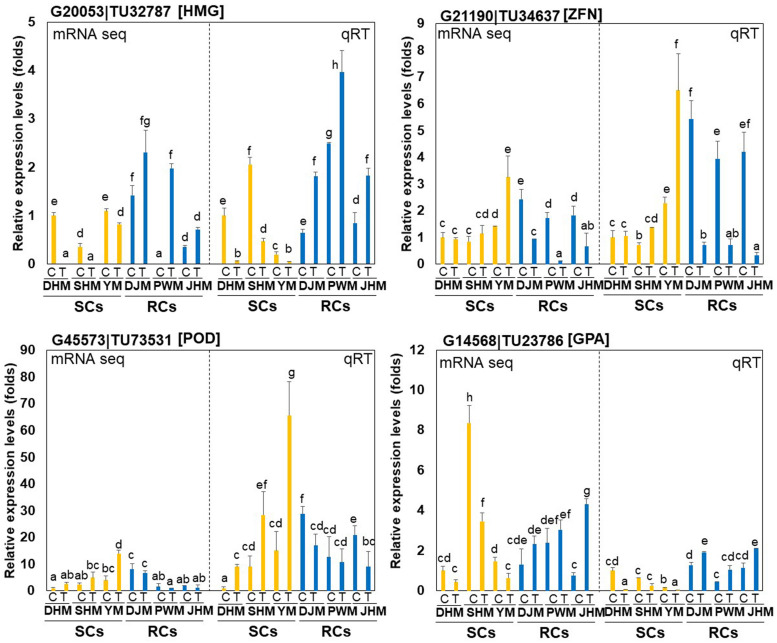
Comparison of quantitative real-time PCR (qRT-PCR) results and RNA-seq data. The graphs show relative transcript levels of two RC-specific genes, hydroxymethylglutaryl-CoA synthase (HMG; G20053| TU32787) and zinc finger protein (ZFN; G21190| TU34637), and two SC-specific genes, cationic peroxidase (POD; G45573| TU73531) and glycerol-3-phosphate acyltransferase (GPA; G14568| TU23786), which were differentially expressed between SCs (yellow bars) and RCs (blue bars) infected with *M. incognita*, as determined by RNA-seq (left *Y*-axis) and qRT-PCR (right *Y*-axis). DHM, Dahomi; DJM, Danjami; PWM, Pungwonmi; SHM, Shinhwangmi; JHM, Juhwangmi; YM, Yulmi; RCs, resistant cultivars; SCs, sensitive cultivars; C, control; T, treatment. Bars denoted with the same letter are not significantly different (*P* = 0.05) according to Dunnett’s test.

We performed qRT-PCR to analyze the expression patterns of four candidate RKN-responsive genes, including two RC-specific genes, hydroxymethylglutaryl-CoA synthase (HMG; G20053| TU32787) and zinc finger protein (ZFN; G21190| TU34637), and two SC-specific genes, cationic peroxidase (POD; G45573| TU73531) and glycerol-3-phosphate acyltransferase (GPA; G14568| TU23786), in the root tissues all six cultivars infected with *M. incognita* (Figure 5). Both qRT-PCR and RNA-seq analysis revealed significant DEGs between the two groups (SCs and RCs). The HMG gene was specifically induced, whereas the ZFN gene specifically downregulated in RCs during RKN infection. On the other hand, the POD gene was specifically induced in SCs during RKN infection, whereas the GPA gene was downregulated.

### Identification and Characterization of DEGs of the Constitutive Defense Response

**FIGURE 6:**
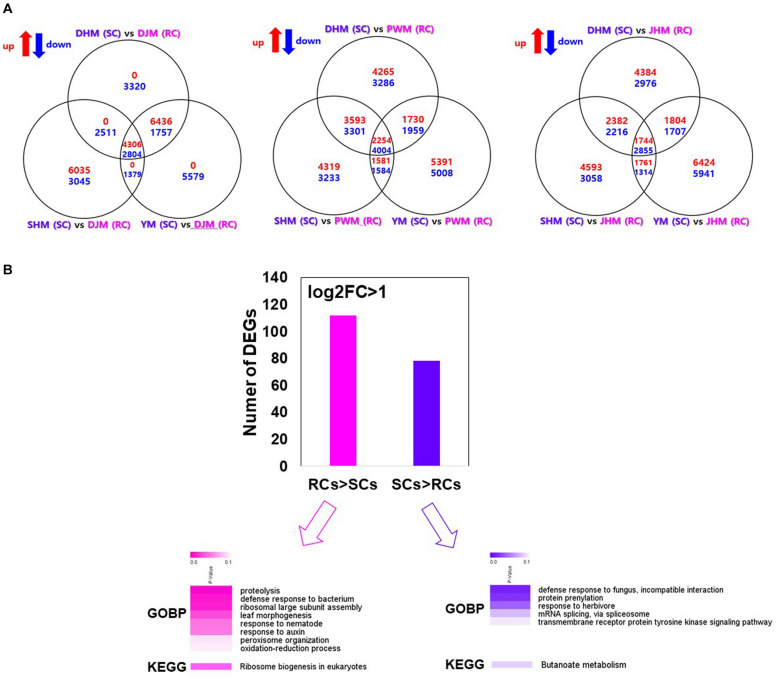
Identification and GO enrichment analysis of sweetpotato genes involved in constitutive defense response against M. incognita infection. **(A)** Venn diagrams showing the number of genes differentially up- or downregulated in pairwise sample comparisons. **(B)** Heatmap of the GO and KEGG enrichment analyses of genes differentially expressed between RCs and SCs. The heatmap shows Benjamini–Hochberg-adjusted (*P* < 0.05) for the biological process GO terms and KEGG pathways associated with DEGs. DHM, Dahomi; DJM, Danjami; PWM, Pungwonmi; SHM, Shinhwangmi; JHM, Juhwangmi; YM, Yulmi.

In RKN-resistant plants, defense related systems are active not only during infection but also under uninfected (normal) conditions (Boots and Best, 2018). Therefore, to confirm the constitutive defense-mediated resistance mechanism in RCs, we compared the transcriptional responses of RCs and SCs under the untreated (control) condition (Figure 6), and identified putative unique transcripts as reliable DEGs (fold change > 2; Kal’s *z*-test FDR *P* < 0.005) in pairwise sample comparisons (Figure 6A). Among the identified DEGs, 4,360 and 2,804 were upregulated and downregulated, respectively, in DJM (an RC) compared with SCs (DHM, SHM, and YM). In addition, 2,254 and 4,004 were significantly up- and downregulated in PWM, while 1,744 and 2,855 were significantly up- and downregulated in JHM compared with SCs. Finally, 112 genes were significantly upregulated in RCs compared with SCs, whereas 78 genes were significantly upregulated in SCs compared with RCs (Figure 6B and Supplementary Tables 5, 6). To functionally characterize the DEGs, we also identified their encoded products via comparisons with *A. thaliana* protein database, and then performed GO and KEGG pathway enrichment analyses of the annotated genes (with Benjamini–Hochberg-adjusted *P* < 0.05). Several biotic stress related GO terms were enriched in the biological process category, including proteolysis, defense response to bacterium, response to nematode, and response to auxin, in RCs compared with SCs under control conditions. GO terms including defense response to fungus, incompatible interaction, protein prenylation and response to herbivore were enriched in SCs compared with RCs under control conditions.

### Differential Regulation of RKN Resistance-Related Candidate Genes Involved in Constitutive Defense Response

**FIGURE 7:**
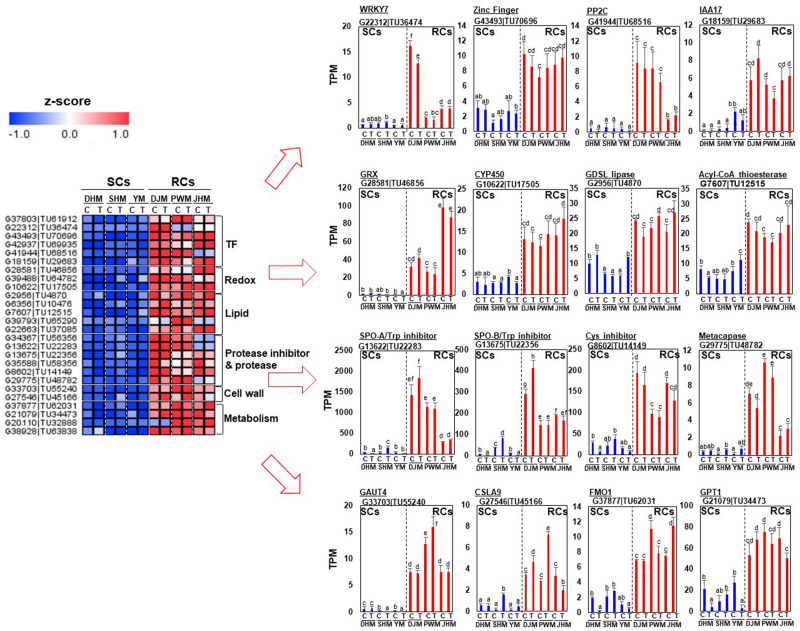
Comparison of the expression levels of RC-specific candidate genes in six sweetpotato cultivars under control and RKN-infected conditions. DEGs related to transcription factors, redox regulation, lipid, cell wall, metabolism, protease and protease inhibitors were identified. The heatmap was constructed using Multi Experiment Viewer (MeV). DHM, Dahomi; DJM, Danjami; PWM, Pungwonmi; SHM, Shinhwangmi; JHM, Juhwangmi; YM, Yulmi; RCs, resistant cultivars; SCs, sensitive cultivars; C, control; T, treatment. Bars denoted with the same letter are not significantly different (*P* = 0.05) according to Dunnett’s test.

**FIGURE 8:**
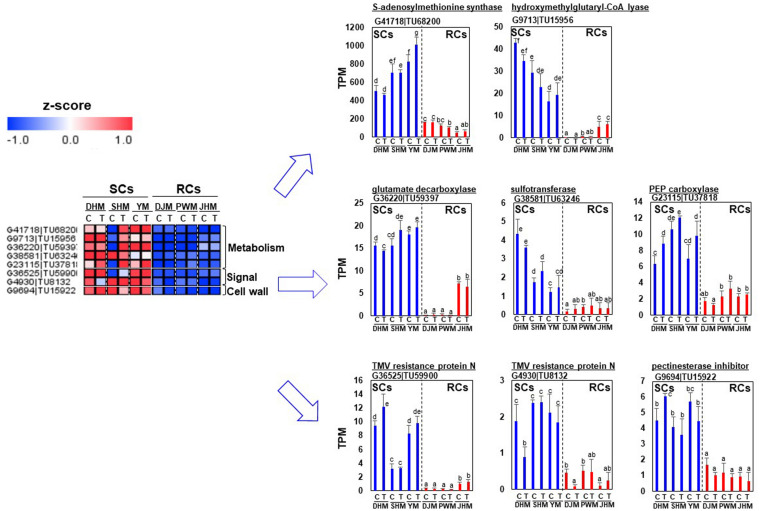
Comparison of the expression levels of SC-specific candidate genes in six sweetpotato cultivars under control and RKN-infected conditions. DEGs related to metabolism, signals and cell wall were identified. The heatmap was constructed using Multi Experiment Viewer (MeV). DHM, Dahomi; DJM, Danjami; PWM, Pungwonmi; SHM, Shinhwangmi; JHM, Juhwangmi; YM, Yulmi; RCs, resistant cultivars; SCs, sensitive cultivars; C, control; T, treatment. Bars denoted with the same letter are not significantly different (*P* = 0.05) according to Dunnett’s test.

First, we identified RC-specific DEGs as candidates for constitutive defense response-related genes (Figure 7). Genes encoding various transcription factors, including putative jasmonic acid (JA)-dependent WRKY7 (G22312| TU36474), a zinc finger protein (G43493| TU70696), auxin repressor IAA17 (G18159| TU29683) and ABA signaling related PP2C (G41944| TU68516), were specifically expressed in RCs under *M. incognita*-infected and control conditions. Genes encoding redox-related GRX (G28581| TU46856), CYP450 (G10622| TU17505), lipid-related GDSL lipase (G2956| TU4870) and acyl-CoA thioesterase (G7607| TU12515) were highly and specifically expressed in RCs. Cell wall and metabolism related genes were also highly expressed in RCs. Interestingly, genes encoding protease inhibitors, including sporamin (G13622| TU22283, G13675| TU22356), which exhibits trypsin inhibitor activity (Cai et al., 2003), and cysteine inhibitor (G8602| TU14149), showed extremely high expression levels in RCs under infected and control conditions. Next, we identified DEGs showing SC-specific expression (Figure 8). Metabolism, signal and cell wall related genes were specifically expressed in SCs under infected and control conditions.

### Temporal Profiling of RKN Resistance Related Genes

**FIGURE 9:**
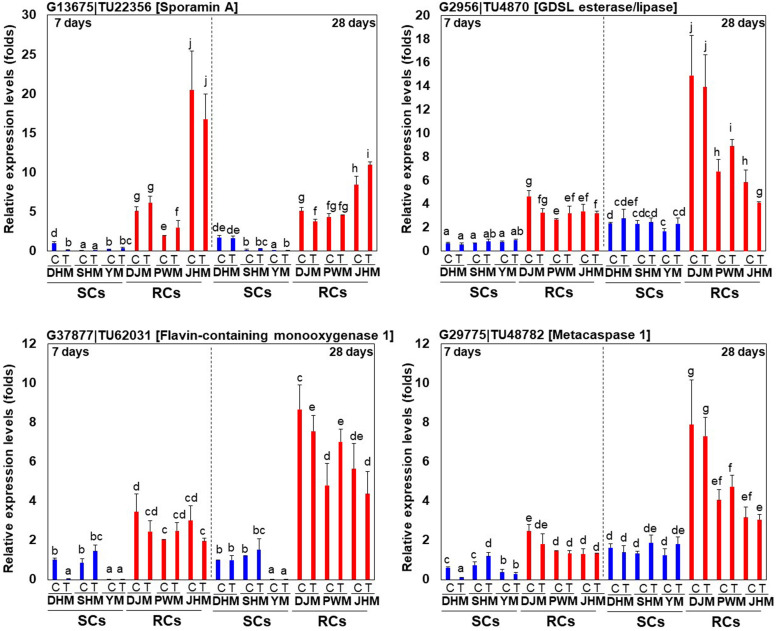
Expression profiling of RC-specific RKN resistance marker genes. Relative transcript levels of DEGs encoding protease inhibitor sporamin A (G13675| TU22356), lipid-related GDSL lipase (G2956| TU4870), secondary metabolism-related flavin-containing monooxygenase G37877| TU62031) and protease metacaspase (G29775| TU48782) in RKN-infected sweetpotato cultivars are shown. DHM, Dahomi; DJM, Danjami; PWM, Pungwonmi; SHM, Shinhwangmi; JHM, Juhwangmi; YM, Yulmi; RCs, resistant cultivars; SCs, sensitive cultivars; C, control; T, treatment. Bars denoted with the same letter are not significantly different (*P* = 0.05) according to Dunnett’s test.

In order to confirm the responses of genes that are specifically regulated in RCs via the constitutive defense response, we investigated for changes in expression at the early (day 7) and late period (day 28) of RKN infection using qRT-PCR (Figure 9). In general, the expression of RC-specific genes, such as those encoding sporamin A (G13675| TU22356), GDSL esterase/lipase (G2956| TU4870), flavin-containing monooxygenase 1 (G37877| TU62031) and metacapase 1 (G29775| TU48782), was upregulated in RCs upon *M. incognita* infection but was downregulated in SCs. Even within RCs, there were changes in the expression of each gene. The expression of sporamin A was lower in all RCs on day 28 after infection than on day 7. By contrast, the expression of GDSL esterase/lipase, flavin-containing monooxygenase 1, and metacapase 1 was higher on day 7 than on day 28 after infection. Interestingly, their levels were the highest in the resistant cultivar DJM. However, the expression of these four genes was higher in RCs than in SCs on both days 7 and 28. Detailed examination of the qRT-PCR data revealed that *M. incognita* infection triggered changes in the expression of RC-specific genes, and the defense mechanism was different between RCs and SCs.

### Differential Regulation of ROS-Related Candidate Genes in Response to RKN

**FIGURE 10:**
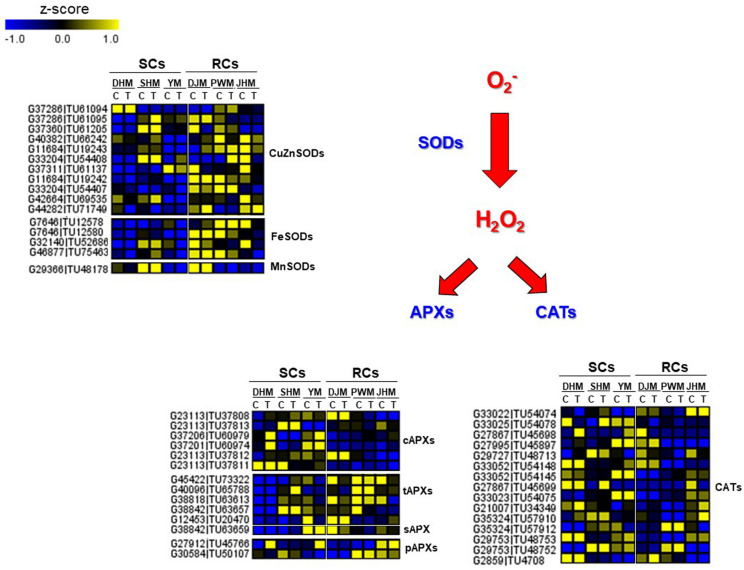
Comparison of the relative expression levels of ROS-related genes in six sweetpotato cultivars under control and RKN-infected conditions. DEGs included the following ROS-related genes. SOD, superoxide dismutase; CuZnSOD, copper zinc SOD; FeSOD, iron SOD; MnSOD, manganese SOD; APX, ascorbate peroxidase; CAT, catalase; cAPX, cytosolic APXs; tAPX, thylakoid APX; sAPX, stroma APX; pAPX, peroxisomal APX. Cultivars were DHM, Dahomi; DJM, Danjami; PWM, Pungwonmi; SHM, Shinhwangmi; JHM, Juhwangmi; YM, Yulmi. Abbreviations are RCs, resistant cultivars; SCs, sensitive cultivars; C, control; T, treatment. The heatmap was constructed using Multi Experiment Viewer (MeV).

During RKN infection, resistance is often associated with hypersensitive response (HR)-mediated programmed cell death (PCD), in which rapid localized cell death in root tissue around the nematode prevents the formation of a developed feeding site. This has been reported for many plant species such as tomato (Williamson, 1999; Melillo et al., 2006), pepper (Pegard et al., 2005), and coffee (Anthony et al., 2005), which show typical HR-mediated PCD during incompatible plant-RKN interactions. At the biochemical level, the rapid generation of reactive oxygen species (ROS), such as superoxide anionic radicals (O_2_^–^) and hydrogen peroxide (H_2_O_2_), is the first reaction in response to attack by avirulent and virulent pathogens. In incompatible interactions between avirulent pathogens and resistant plants, transient ROS production is followed by massive and prolonged ROS accumulation, and the latter is intimately associated with the HR response (De Gara et al., 2003). These two-phase kinetics of ROS production are typical of the incompatible defense mechanism of HR-mediated PCD. ROS play an important role in plant defense, and during pathogen attack ROS, such as H_2_O_2_, are generated by the enzyme superoxide dismutases (SODs), and H_2_O_2_ detoxifying enzymes such as ascorbate peroxidase (APX) and catalase (CAT) are often suppressed in pathogen resistant plants (Klessig et al., 2000). As a result, plants produce more ROS and interactions with these components lead to a HR-mediated defense response in plant cells. Particularly, the H_2_O_2_ plays a major role in triggering HR-mediated defense mechanisms in incompatible interactions between plants and pathogens including RKN (Dangl and Jones, 2001; Melillo et al., 2006). In this study, we identified DEGs that are specifically involved in the regulation of ROS (H_2_O_2_) generation during the defense response in RCs and SCs (Figure 10). In RCs, genes encoding H_2_O_2_ generating SODs were expressed at higher levels than in SCs, whereas genes encoding H_2_O_2_ scavenging cytosolic APXs (cAPXs) and CATs were expressed at lower levels in RCs than in SCs, which could indicate that RCs generate more H_2_O_2_ than SCs. These DEG patterns correlated with phenotypic responses in sweetpotato (Figure 1).

## Discussion

Defense against pathogens, including plant parasitic nematodes, results from a complex set of interdependent mechanisms, ranging from mechanical and chemical barriers to the complex array of initial effector molecules in the plant immune system (Boots and Best, 2018). Preformed elements of defense, such as cell wall modification, toxic metabolites and phytochemicals, represent the first barrier for any kind of pathogenic invader. The complicated induced defense response system, which is activated upon infection, is based on the capability of plants to recognize and identify pathogenic invaders including plant parasitic nematodes. During pathogen infection, plants exhibit resistance by altering the global transcriptional responses to activate induced and/or constitutive defense. Plant parasitic nematodes induce a wide range of resistance responses in plants, with some interactions resulting in a qualitative response, which involves induced and/or constitutive defense. However, the molecular mechanisms of nematode resistance in sweetpotato are poorly understood. Although multiple resistance genes involved in qualitative response to nematode infection have been discovered in sweetpotato through transcriptome, proteome and transgenic analyses, the exact function and mode of action of these genes remain unknown (Fan et al., 2015; Zhai et al., 2016; Ha et al., 2017; Lee et al., 2019). Here, we performed a comprehensive analysis of gene expression in three RCs and three SCs of sweetpotato before and after infection with *M. incognita* infection to characterize induced and constitutive defense responses.

During the induced defense response, different resistance related genes can function in different ways in the contrasting sweetpotato cultivars. When examining gene expression data, the majority of the known resistance genes, such as those encoding ethylene biosynthesis-related ACC oxidases (G2553| TU4254, G3204| TU5260, and G20891| TU34171), polyprenol reductase (G18703| TU30556), trehalose 6-phosphate phosphatases (G42399| TU69169 and G42693| TU69581) and cationic peroxidases (G41119| TU67300 and G45573| TU73531), which are upregulated, especially in SCs, upon nematode infection, are often the central focus, as pathogen resistance pathways are expected to be induced (Figure 4 and Supplementary Table 3; Bajda et al., 2009; Melillo et al., 2014; Zhang et al., 2016; Leonetti et al., 2017; Sung et al., 2019). There can also be considerable value in genes downregulated upon RKN infection, especially in SCs, which may explain RKN success (Figure 4 and Supplementary Table 4) as noted in other studies (Guimaraes et al., 2015; Shukla et al., 2018).

With respect to the constitutive defense response, we identified 177 genes uniquely upregulated in RCs compared with SCs under untreated control conditions (Figure 6 and Supplementary Table 5). Notably, we identified JA- or ethylene-dependent genes encoding WRKY7 (G22312| TU36474), CYP450 (G19945| TU32621, G39488| TU64782, and G10622| TU17505), GDSL lipase (G6356| TU10476 and G2956| TU4870), sporamin (G13622| TU22283, G13675| TU22356, and G34367| TU56356) and metacaspase (G29775| TU48782), indicating that the pathways involving these genes might be important for nematode defense (Wang et al., 2000, 2002; Kim et al., 2006; Kandel et al., 2007; Kwon et al., 2009; Rajendran et al., 2014; Liu et al., 2016). Interestingly, these genes were upregulated in RCs but downregulated in SCs during *M. incognita* infection (Figures 7, 9). However, the involvement of these genes in nematode resistance is not verified and needs further investigation.

**FIGURE 11:**
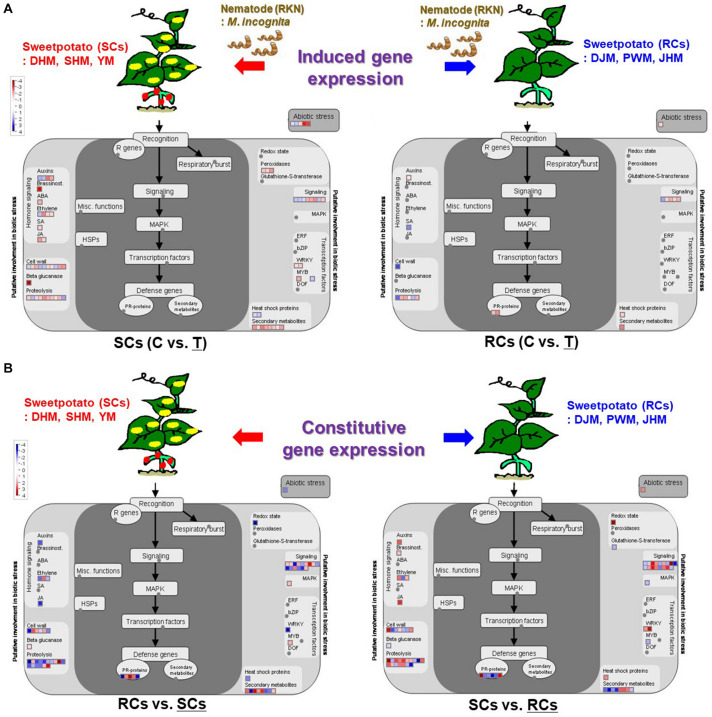
MapMan diagram of sweetpotato genes involved in induced defense and constitutive defense response to *M. incognita* infection in SCs (left) and RCs (right). **(A)** Induced defense response to *M. incognita* infection in sweetpoato. **(B)** Constitutive defense response to *M. incognita* infection in sweetpoato. An overview of gene expression patterns (log_2_FC) in infected plants relative to the untreated control is shown. Dots indicate the different paralogous genes encoding proteins related to a certain step in the defense response. Red dots indicate upregulation, blue dots indicate downregulation and gray dots indicate no response. DHM, Dahomi; DJM, Danjami; PWM, Pungwonmi; SHM, Shinhwangmi; JHM, Juhwangmi; YM, Yulmi; RCs, resistant cultivars; SCs, sensitive cultivars; C, control; T, treatment.

Figure 11 show genes involved in the response to nematode infection including those related to pathogen recognition, defense response signaling, phytohormones, cell wall metabolism, proteolysis, redox state, transcription factors and secondary metabolism. Overall, the MapMan ontology analysis enabled us to construct a genome-wide outline of the expression of sweetpotato genes that respond to *M. incognita* infection by identifying pathways involved in the main steps leading to induced and constitutive defense responses. During the induced defense response, genes related to phytohormones, cell wall and proteolysis-mediated response were upregulated in SCs during *M. incognita* infection (Figure 11A). Additionally, genes related to beta-glucanase, peroxidase and secondary metabolites were also upregulated. However, in RCs, genes related to phytohormones, cell wall, proteolysis, redox regulation and abiotic stress were downregulated upon *M. incognita* infection. These data suggest that SCs respond to RKN infection, whereas RCs do not exhibit induced defense response upon RKN infection. In the case of constitutive defense response, interestingly, genes involved in phytohormones (auxin, ethylene, and JA) and proteolysis-related defense signaling pathways (such as protease inhibitor and protease), which are directly or indirectly related to resistance responses and play a role in plant immunity, were upregulated in RCs under control conditions (Figure 11B).

Previously, we reported the proteome and transcriptome profiling of two sweetpotato cultivars, namely JHM and YM, at different temporal points after infection with the RKN *M. incognita* (Ha et al., 2017; Lee et al., 2019). The results showed that JHM was more resistant to RKN infection than YM when plants were cultivated for 50 days in a growth chamber and 90 days under greenhouse conditions. A proteomic study of 50-day cultivated sweetpotato, confirmed differences in the intensities of 64 protein spots on 2-D gel electrophoresis gels between the two cultivars during RKN infection (Ha et al., 2017). Of these 64 protein spots, 20 were identified as belonging to widely different functional categories, such as the defense response, cell structure, and energy metabolism. In a transcriptomic study of 90-day cultivated sweetpotato, 74,733 transcripts were assembled and a number of unique genes were found to be differentially expressed upon RKN infection (Lee et al., 2019). DEGs encoding transcription factors involved in various hormonal signaling-related pathways were identified as being associated with RKN infection. SA-dependent WRKY genes were not expressed or were slightly induced upon RKN infection in both cultivars. By contrast, the expression of ET-dependent ERF and JA-dependent MYC genes was more upregulated in JHM than in YM during RKN infection. Various pathogenesis-related (PR) genes activated through transcription factor dependent pathways were also regulated during RKN infection. Previous transcriptome and proteomic analysis were limited in their ability to elucidate the mechanism of RKN resistance in sweet potato because the analysis was limited to only two representative cultivars, YM and JHM. In this study, six cultivars from different origins were used and the study was conducted by dividing the cultivars into two groups according to susceptibility to RKN, namely RCs (DHM, PWM, and JHM) and SCs (DJM, SHM, and YM). Transcriptomics examined induced and constitutive defense response-related transcriptional changes in these cultivars 7 days after inoculation with RKN, which revealed transcriptional changes in genes involved in the induced defense response and constitutive defense during RKN infection. This study is the first to study to examine the common resistance and susceptibility mechanisms of sweetpotato using cultivars of different origins.

## Conclusion

In conclusion, we identified changes in the expression of defense response-related genes in a total of six sweetpotato cultivars sensitive or resistant to *M. incognita* infection, thus characterizing the induced and constitutive defense response mechanisms. We identified many candidate genes that might trigger changes in specific induced and constitutive defense responses involved in phytohormone regulation, defense related metabolism and RKN signaling in sweetpotato. The identification of RKN resistance-related genes by marker-assisted selection could offer several advantages for nematode control in an integrated management system. Further investigation is needed to elucidate the exact role of each candidate gene in the regulation of the signaling pathway involved in the induced and/or constitutive defense response of sweetpotato during infection with RKN. Transgenic plants overexpressing or underexpressing each candidate gene will be generated to determine their roles in RKN-resistant mechanisms. Overall, our results provide valuable information for the development of crops with enhanced resistance to RKNs.

## Data Availability Statement

The original contributions presented in the study are publicly available. This data can be found here: The RNA-seq data has been uploaded to NCBI (https://www.ncbi.nlm.nih.gov/). The BioProject ID is PRJNA429283.

## Author Contributions

Y-HK, I-HL, DS, and HK conceived and designed the experiments. I-HL, HK, and J-WY performed the experiments. KN and K-LL analyzed the data. S-SK and JL contributed to the analyses and provided materials and reagents. Y-HK, I-HL, DS, and HK wrote the manuscript. All authors contributed to the article and approved the submitted version.

## Conflict of Interest

The authors declare that the research was conducted in the absence of any commercial or financial relationships that could be construed as a potential conflict of interest.

## Footnotes

1 https://ipomoea-genome.org/

2 https://david.ncifcrf.gov/

3 https://mapman.gabipd.org/home

4 https://ipomoea-genome.org/

## Supplementary Material

Supplementary Figure 1 Volcano plots showing pairwise comparisons of transcript levels across samples. Log_2_ fold change (logFC) between two samples is plotted on the *Y*-axis, and log_2_ average of the counts normalized by size factor is shown on the *X*-axis. Red dots indicate transcripts with logFC significantly >2 or lower than –2. Black dots indicate transcripts with logFC was between –2 and 2. DHM, Dahomi; DJM, Danjami; PWM, Pungwonmi; SHM, Shinhwangmi; JHM, Juhwangmi; YM, Yulmi; RCs, resistant cultivars; SCs, sensitive cultivars; C, control; T, treatment.
