# Supplementary figures and images for: The Defense Response Involved in Sweetpotato Resistance to Root-Knot Nematode Meloidogyne incognita: Comparison of Root Transcriptomes of Resistant and Susceptible Sweetpotato Cultivars With Respect to Induced and Constitutive Defense Responses
Source: Front Plant Sci. 2021 May 5;12:671677. doi: 10.3389/fpls.2021.671677 (PMC8131533; doi:10.3389/fpls.2021.671677)

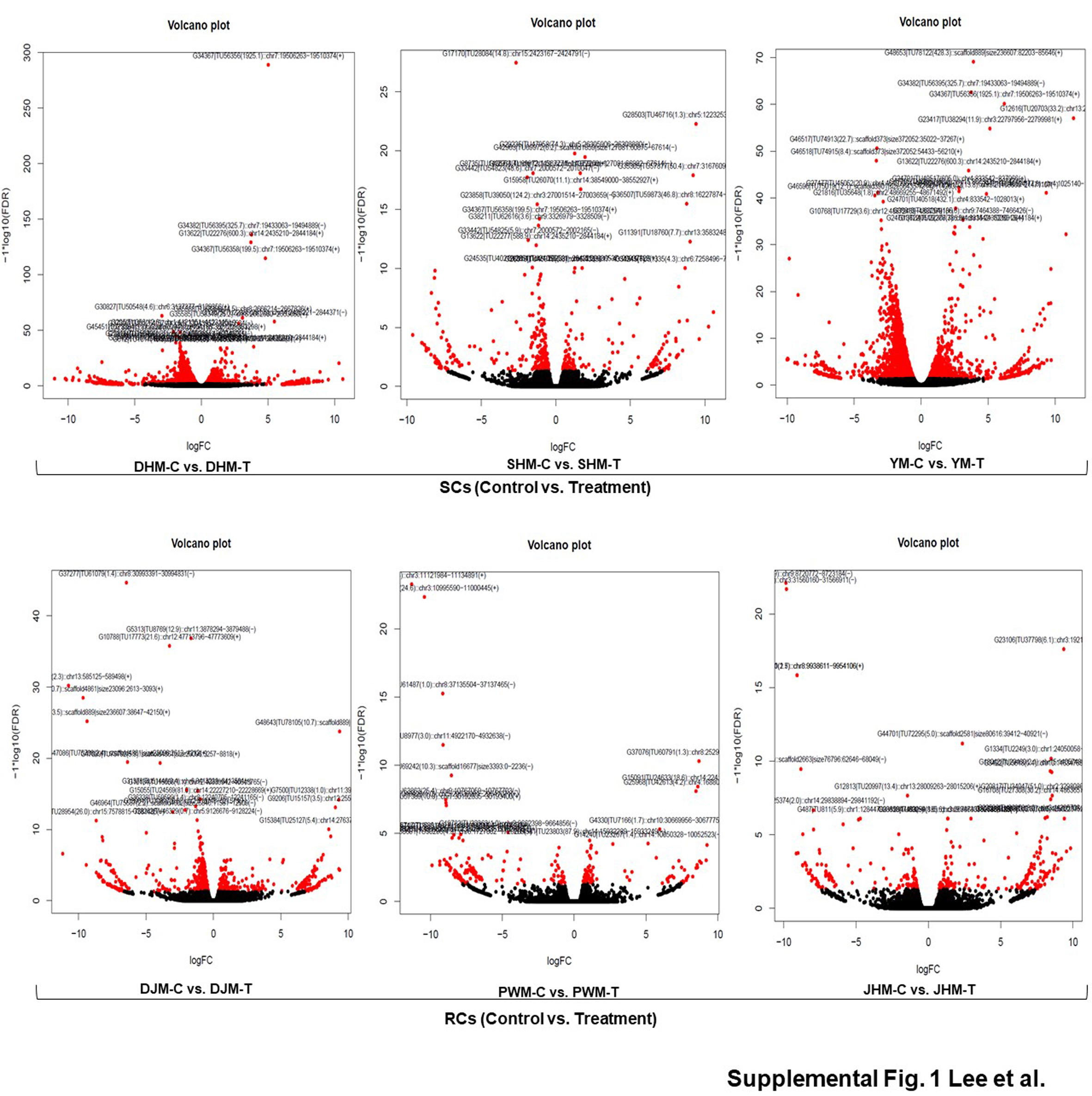

Supplement: Supplementary Figure 1 — Volcano plots showing pairwise comparisons of transcript levels across samples. Log2 fold change (logFC) between two samples is plotted on the Y-axis, and log2 average of the counts normalized by size factor is shown on the X-axis. Red dots indicate transcripts with logFC significantly >2 or lower than –2. Black dots indicate transcripts with logFC was between –2 and 2. DHM, Dahomi; DJM, Danjami; PWM, Pungwonmi; SHM, Shinhwangmi; JHM, Juhwangmi; YM, Yulmi; RCs, resistant cultivars; SCs, sensitive cultivars; C, control; T, treatment. [file Image_1.JPEG]
